# Supplementary material for: Legionella pneumophila and Free-Living Nematodes: Environmental Co-Occurrence and Trophic Link
Source: Microorganisms. 2023 Mar 13;11(3):738. doi: 10.3390/microorganisms11030738 (PMC10056204; doi:10.3390/microorganisms11030738)
Supplement: Supplementary file 1 [file microorganisms-11-00738-s001.zip › Table S1.pdf]

TABLE S1 Location and water temperature [°C] of swimming lakes in Berlin-Brandenburg investigated during the field campaign in summer 2018. The lakes were only sampled when biofilm was detected (+ detection, - no detection).

Name	Region	Latitude	Longitude	Water temperature	Biofilm detection	Reference
Dewinsee	Brandenburg	52°44′30.6′′N	13°38′46.0′′E	N/A	-	
Flakensee	Brandenburg	52°25′59.0′′N	13°45′55.9′′E	20.7	-	1
Flughafensee	Berlin	52°34′06.6′′N	13°17′09.0′′E	27.7	+	2
Gorinsee	Brandenburg	52°41′16.0′′N	13°28′06.5′′E	27.7	-	3
Großer Wukensee	Brandenburg	52°46′27.1′′N	13°36′22.8′′E	27.7	-	3
Jungfernheideteich	Berlin	52°32′37.5′′N	13°16′36.2′′E	N/A	-	
Kalksee	Brandenburg	52°27′30.1′′N	13°45′54.8′′E	21.5	+	1
Kiessee Schildow	Brandenburg	52°39′10.3′′N	13°22′58.5′′E	23.3	+	4
Krumme Lanke	Berlin	52°27′06.0′′N	13°13′54.6′′E	27.1	-	5
Motzener See	Brandenburg	52°12′49.6′′N	13°34′28.0′′E	N/A	-	
Mühlenbecker See	Brandenburg	52°41′29.7′′N	13°23′43.1′′E	N/A	-	
Mühlenteich	Brandenburg	52°41′41.9′′N	13°24′40.9′′E	N/A	-	
Obersee	Brandenburg	52°45′38.2′′N	13°33′02.8′′E	26.7	-	3
Pätzer Hintersee	Brandenburg	52°12′29.8′′N	13°37′52.2′′E	N/A	-	
Plötzensee	Berlin	52°32′37.4′′N	13°19′50.9′′E	27.6	-	6
Quarry pond Arkenberge	Berlin	52°38′15.3′′N	13°24′37.1′′E	N/A	+	
Quarry pond Biesdorf	Berlin	52°30′11.5′′N	13°32′54.7′′E	26.6	+	7
Rahmer See	Brandenburg	52°45′01.0′′N	13°25′01.2′′E	22.8	+	4
Schlachtensee	Berlin	52°26′35.7′′N	13°13′03.9′′E	22.0	-	8
Stolzenhagener See	Brandenburg	52°45′43.8′′N	13°26′14.0′′E	26.6	+	3
Summter See	Brandenburg	52°41′35.0′′N	13°22′20.7′′E	N/A	-	
Tegeler See	Berlin	52°34′33.5′′N	13°15′23.8′′E	23.0	-	8
Wandlitzer See	Brandenburg	52°45′23.0′′N	13°28′02.9′′E	26.6	+	3
Wannsee	Berlin	52°26′14.2′′N	13°10′18.6′′E	N/A	-	
Weißer See	Berlin	52°33′17.2′′N	13°27′47.8′′E	24.0	+	8
Zeesener See	Brandenburg	52°16′12.1′′N	13°38′48.8′′E	N/A	-	

References

1. https://www.landkreis-oder-spree.de/media/custom/2689_1595_1.PDF?1530869752 [Accessed: 20.10.2022]
2. https://wasserportal.berlin.de/station.php?anzeige=tg&sgrafik=g&stable=ows&sstation=5800303 [Accessed: 20.10.2022]
3. https://presse.barnim.de/documents/tabelle-badewasserqualitaet-80833 [Accessed: 20.10.2022]
4. https://hohen-neuendorf.de/de/stadt-leben/aktuelles/badewarnungen-fuer-drei-seen [Accessed: 20.10.2022]
5. https://wasserportal.berlin.de/station.php?anzeige=tg&sgrafik=g&stable=ows&sstation=5800107 [Accessed: 20.10.2022]
6. https://wasserportal.berlin.de/station.php?anzeige=tg&sgrafik=g&stable=ows&sstation=5800312 [Accessed: 20.10.2022]
7. https://wasserportal.berlin.de/station.php?anzeige=tg&sgrafik=g&stable=ows&sstation=5800317 [Accessed: 20.10.2022]
8. https://www.morgenpost.de/berlin/article214942137/Sportlich-schick-romantisch-Badeseen-fuer-jeden-Typ.html [Accessed: 20.10.2022]
